# Photodynamic Therapy in Cancer: Insights into Cellular and Molecular Pathways

**DOI:** 10.3390/cimb47020069

**Published:** 2025-01-21

**Authors:** Vincenzo Papa, Fabiana Furci, Paola Lucia Minciullo, Marco Casciaro, Alessandro Allegra, Sebastiano Gangemi

**Affiliations:** 1 Allergy and Clinical Immunology Unit, Department of Clinical and Experimental Medicine, University of Messina, Via Consolare Valeria, 98125 Messina, Italy; papavi.994@gmail.com (V.P.); pminciullo@unime.it (P.L.M.); gangemis@unime.it (S.G.); 2 Provincial Healthcare Unit, Section of Allergy, 89900 Vibo Valentia, Italy; fabianafurci@gmail.com; 3 Division of Hematology, Department of Human Pathology in Adulthood and Childhood “Gaetano Barresi”, University of Messina, Via Consolare Valeria, 98125 Messina, Italy; aallegra@unime.it

**Keywords:** photodynamic therapy, cancer, cytomolecular mechanisms, inflammation, oxidative stress, stress response molecules, cytokines, nanomedicine, immunotherapy

## Abstract

Photodynamic therapy is a non-ionizing radiation treatment that utilizes a photosensitizer in combination with light to produce singlet oxygen. This singlet oxygen induces anti-cancer effects by causing apoptotic, necrotic, or autophagic cell death in tumor cells. Currently, photodynamic therapy is employed in oncology to treat various cancers. In the presence of oxygen, this non-invasive approach leads to direct tumor cell death, damage to microvasculature, and the induction of a local inflammatory response. These effects allow photodynamic therapy to be effective in treating early-stage tumors, extending survival in cases where surgery is not feasible, and significantly improving quality of life. In this paper, we provide a state of the art on cytomolecular mechanisms and associated pathways involved in photodynamic therapy. By integrating these mechanistic insights with the most recent advancements in nanotechnology, this phototherapeutic approach has the potential to become a prevalent treatment option within conventional cancer therapies, enhancing its application in precision medicine.

## 1. Introduction

Photodynamic therapy (PDT) is a promising non-invasive phototherapeutic approach widely studied in oncology, with applications extending to other clinical settings. Its origins date back to 1900 [1]. The procedure involves administering a photosensitizing pro-drug, which, when photoactivated by light of a specific wavelength (ideally between 650 and 850 nm) in the presence of oxygen, triggers the formation of reactive oxygen species (ROS). These ROS are highly cytotoxic to cells where the photosensitizer (PS) accumulates [2].

Biochemically, PDT operates through two types of reactions (Type 1 and Type 2) initiated when the PS absorbs a photon and is electronically excited to a singlet state. The excited PS transitions to a triplet state via intersystem crossing. In a Type 1 reaction, an electron or hydrogen is transferred from the triplet state of the PS to nearby biomolecules or directly to oxygen, resulting in the generation of radical species such as hydrogen peroxide, hydroperoxyl, nitric oxide, superoxide ions, and hydroxyl radicals. Alternatively, in a Type 2 reaction, there is direct energy transfer from the excited PS to molecular oxygen, leading to the production of singlet oxygen. Such direct transfer occurs when PS and molecular oxygen have aligned electronic spins. The latter is a property of molecules that determines their quantum state [2,3]. Singlet oxygen is highly reactive towards biomolecules, but its intracellular lifetime does not exceed 15–30 μs, and its propagation distance is up to 0.5–1 μm. On the other hand, among the various ROS produced, hydrogen peroxide, a precursor of the hydroxyl radical, has a longer intracellular lifetime (up to 1 ms) and, in addition to inducing the oxidation of membrane lipids and proteins, plays a regulatory role by interacting with proteins involved in various cellular signaling pathways, thus triggering either cytoprotective mechanisms or cell death processes [4].

In this context of redox reactions, most of the reactive species formed interconvert with each other; furthermore, both radicals and singlet oxygen can attack PS, leading to its degradation [5]. An important consideration is the influence of the intracellular localization of PS in determining therapeutic efficacy and the type of cytomolecular events elicited by PDT [6]. More specifically, photodamage to the endoplasmic reticulum induces a massive intracellular calcium release, which plays a major role in triggering necroptosis. Additionally, photodamage to other organelles such as mitochondria and lysosomes is equally crucial in inducing various cell death mechanisms. Beyond the well-known induction of the apoptotic process (via cytoplasmic release of cytochrome c), the mitochondrial localization of PS also induces necrosis and autophagy depending on the PDT dose (which is defined by PS concentration and light intensity). Specifically, high PDT doses induce necrosis, mild PDT doses induce apoptosis, and low PDT doses induce autophagy as a cytoprotective mechanism for mitochondrial repair (“mitophagy”), which, if unsuccessful, leads to autophagic cell death. Similarly, lysosomal photodamage depends on the PDT dose; high PDT doses cause complete organelle destruction with massive release of lysosomal enzymes into the cytoplasm and induction of necrosis. On the other hand, partial lysosomal damage results in the release of hydrolases, which induce apoptosis and autophagic cell death [5].

PDT offers several advantages, including its curative potential, especially in early-stage tumors, and its ability to extend survival in cases of inoperable cancers, thereby improving quality of life.

Additional factors that support the use of this therapy include its low tissue toxicity, minimal systemic reactions, significant reduction in long-term morbidity, and excellent cosmetic outcomes, as well as the preservation of organ function [7].

The biological behavior of cancer is characterized by mechanisms that ensure its survival, primarily through two crucial processes: tumor neoangiogenesis and evasion of immune surveillance. Tumors achieve this evasion by presenting or secreting molecules that inhibit the immune response [8]. PDT offers an advantage over conventional therapies by targeting these two distinct biological processes. Specifically, PDT’s ability to induce cell death through direct damage is complemented by its targeting of neoangiogenesis. The accumulation of PSs within the endothelial cells leads to indirect cytotoxic effects that destroy the tumor vasculature, causing hypoxia, nutrient deprivation, and accumulation of toxic metabolites, which collectively trigger cell death. Additionally, PDT-induced direct cytotoxicity can stimulate the release of inflammatory cytokines and alarmins into the bloodstream, thereby eliciting an innate immune response that is further enhanced by the activation of a sustained adaptive immune response [8,9,10]. Among the major PSs being commercially approved for clinical use are m-tetrahydroxyphenylchlorin (mTHPC), porfimer sodium (Photofrin), hemoporfin, 5-aminolevulinic acid (5-ALA), methylamine levulinate hydrochloride (MAL), verteporfin, talaporfin, hexylamine levulinate hydrochloride (HAL), chlorin e6 (Ce6), foscan (Temoporfin), 3-Devinyl-3-(1′-hexyloxyethyl) pyrylporphyrin-a (HPPH), and pheophorbide a [3,11].

Understanding the molecular and therapeutic mechanisms of PDT suggests that combining PDT with other therapeutic strategies could enhance the overall therapeutic index of treatments in modern oncology.

Based on this premise, we conducted a narrative review of the current state of the art regarding the cytomolecular mechanisms modulated by PDT in various neoplastic pathologies. Moving into basic research, this paper highlights how understanding these mechanisms, particularly when supported by the new frontiers of combination therapy in nanomedicine, is steering medicine towards a more precision-focused approach.

Search Strategy: we performed a comprehensive search in PubMed electronic database for relevant cytomolecular studies, without restrictions on study type.

Inclusion Criteria: only English-language papers from the past decade that elucidate pathways directly influenced by PDT were included.

Exclusion Criteria: papers addressing comparative efficacy studies, as well as cytomolecular mechanisms resulting from the combination of PDT with other anti-cancer therapeutic strategies, were excluded.

## 2. Breast Cancer

Breast cancer is the most prevalent form of cancer and the leading cause of cancer-related mortality worldwide. It predominantly affects women, with its incidence increasing with age. Modifiable risk factors include obesity, alcohol consumption, physical inactivity, and hormone therapy, while non-modifiable factors encompass age, genetic predisposition, and endogenous hormonal exposure. Protective factors include breastfeeding, physical activity, and the use of aspirin and nonsteroidal anti-inflammatory drugs. Tumorigenesis is rooted in the dysregulation of pathways controlling apoptosis and cellular proliferation [12].

In recent decades, PDT has emerged as a promising novel approach for the treatment of this malignancy, proving to be effective, minimally invasive, and associated with negligible adverse reactions. In clinical settings, its application has been proposed for managing breast cancers refractory to standard treatments, with the added potential of restoring cancer cell sensitivity to conventional therapies [13]. The mechanisms triggered by PDT include tumor cell killing via direct phototoxic damage, leading to cell death through apoptosis, necrosis, or autophagy. Additionally, PDT induces vascular damage within the tumor and stimulates host immunity, resulting in an antitumor inflammatory response [13]. More specifically, the type of cell death induced by PDT depends on the intracellular localization of the PS. Mitochondrial or cytoplasmic localization of PS triggers apoptosis, activating the intrinsic mitochondrial pathway through cytochrome c (cyt-c) release or damaging the nuclear factor kappa B (NFĸB) pathway when localized in the cytoplasm. Endoplasmic reticulum (ER) localization induces autophagy via the activation of Beclin-1 and the mechanistic target of rapamycin (mTOR) protein. Necrosis, on the other hand, occurs following the disruption of the cell membrane when PS localizes in this region. Indirect cytotoxicity results from PS uptake and activation in endothelial cells of the tumor vasculature, leading to vascular destruction. This deprives tumor cells of essential oxygen and nutrients while causing metabolic waste accumulation, culminating in cell death [9]. Following PDT-induced cytotoxicity (both direct and indirect), the release of cytokines and tumor necrosis factors (TNFs) triggers an antitumor inflammatory response and immunogenic cell death (ICD) of cancer cells. Interestingly, the activation of the innate immune response, followed by the adaptive immune response, leads to the development of memory T cells and so to a long-lasting antitumor immunity which can effectively control metastasis and prevent tumor recurrence after PDT [9]. Dos Santos et al. evaluated the efficacy of methylene blue-mediated PDT (MB-PDT) in three breast epithelial cell lines, both tumorigenic and non-tumorigenic, demonstrating its ability to selectively induce massive tumor cell destruction. Moreover, they observed that apoptosis does not appear to be the predominant cell death pathway triggered by MB-PDT, not even through a caspase-independent mechanism. Interestingly, autophagy emerges as an initial pro-survival response to oxidative damage induced by MB-PDT in triple-negative breast cancer (TNBC) cells, but not in luminal A cells [14].

Cutting-edge is the emerging research on the PDT potential to modulate breast cancer epigenetics in an antitumor direction, specifically by suppressing the expression of oncogenic microRNAs (miRNAs) and upregulating tumor-suppressing ones. Notably, the PDT-induced suppression of oncogenic miR-21, miR-221, and miR-27a in female Wistar rats—miRNAs typically overexpressed in this malignancy—paves the way for further in vivo studies on the subject [15].

Moreover, an in-depth investigation into the effects of PDT on breast cancer epigenetics could elucidate novel mechanisms of tumor cell survival directly linked to PDT-induced oxidative damage and the consequent impaired biogenesis and maturation of tumor-suppressor miRNAs. This has been preliminarily observed for Hsa-miR-16 in a TNBC cell model treated with δ-aminolevulinic acid (ALA)-PDT [16]. On the other hand, the latest nanotechnological optimizations of ALA-PDT in breast cancer cells highlight the efficacy of this approach in inhibiting the phosphatidylinositol 3-kinase (Pi3K)/protein kinase B (AKT)/mTOR signaling pathway, a key cellular survival mechanism, thereby inducing mitochondrial apoptotic events [17].

Highlights:

- Apoptosis (primarily caspase-dependent), necrosis, autophagy and immunogenic cell death are the main PDT-induced cell death pathways in breast cancer.
- The presumed role of autophagy as an initial survival mechanism has been revealed.
- Type of cell death is strictly dependent on the intracellular PS localization.
- Potential epigenetic regulatory role of PDT through stimulation of oncosuppressive miRNAs and downregulation of tumorigenic ones would be emerging.
- PDT impacts Pi3K/AKT/mTOR signaling, a crucial survival pathway.

## 3. Skin Cancer

Skin cancer is the most frequently diagnosed form of cancer worldwide. The most prevalent types include keratinocyte carcinomas, such as squamous cell carcinoma (SCC) and basal cell carcinoma (BCC), as well as cutaneous melanoma. Since 1990, these cancers have shown a global increase in prevalence and disability-adjusted life years, despite ongoing prevention efforts, particularly those aimed at precise risk stratification [18,19].

Within a context of an even greater need for novel therapeutic strategies, the clinical application of PDT offers significant advantages, including precise targeting, minimal trauma, synergy with conventional treatments, and repeatability. Currently, PDT is an established alternative treatment for non-melanoma skin cancers (BCC and SCC), potentially applicable in neoadjuvant settings. Additionally, a growing body of literature supports its effectiveness in the treatment of superficial and localized forms of cutaneous melanoma [20,21].

The promising efficacy of PDT relies on the elicitation of three distinct phenomena: cytotoxicity, microvascular killing, and immune response activation. The cytotoxic effect stems from ROS production, leading to the induction of apoptosis, necrosis, and ferroptosis as cell death pathways. Among these, mitochondria-dependent apoptosis, characterized by cyt c release and subsequent downstream events, is considered the main mechanism of cell death. Necrosis, on the other hand, arises from the activation of receptor-interacting protein kinase 1 (RIPK1), lysosomal damage, and intracellular calcium overload [22].

Specifically, massive ROS production is thought to elicit necrosis, whereas lower oxidative stress (OS) primarily induces apoptosis as the main mechanism of cell death [22,23,24]. Molecularly, in SCC, the antiproliferative effect of topical ALA-PDT is primarily attributed to the inhibition of Signal transducer and activator of transcription 3 (STAT3) expression [25].

In parallel, PDT-induced ICD occurs through the release of damage-associated molecular patterns (DAMPs) or alarmins such as heat shock protein (HSP)-70, HSP-90, High-Mobility Group Box 1 (HMGB1), and Interleukin (IL)-1β. The microvascular killing effect is mediated by direct damage to endothelial cells, resulting in endothelial necrosis. Noteworthy is the PDT-induced antitumor immune response, manifested as an acute local inflammatory reaction that is sustained or amplified through the concomitant inhibition of anti-inflammatory cytokines, such as IL-10 and transforming growth factor (TGF)-β. Interestingly, in BCC patients treated with PDT, an increased immunoreactivity of circulating leukocytes against the BCC-related tumor antigen Hedgehog-interacting protein 1 (Hip-1) has been observed. Similarly, preliminary evidence suggests the immunogenic potential of ALA-PDT in SCC through the increased release of DAMPs [22,25]. Concerning cutaneous melanoma, its high resistance to conventional therapies makes PDT a promising treatment strategy. Valli et al. investigated the effectiveness of cationic zinc (II) phthalocyanine Pc13 as a PS in various melanoma cell lines. Pc13 treatment activates several pathways, including Mitogen-activated protein kinase (MAPK) p38, Extracellular signal-regulated kinase (ERK), c-Jun N-terminal kinase (JNK), and the PI3K/AKT. Pc13 irradiation also triggers an autophagic response, evidenced by increased levels of Beclin-1, LC3-II, and generating green fluorescent protein (GFP)-LC3 punctate staining. Autophagy is promoted by JNK and negatively regulated by the PI3K/AKT pathway. Blocking autophagy enhances Pc13 phototoxicity, suggesting that this event protects against apoptotic cell death. Moreover, repeated cycles of Pc13 PDT in A375 cells led to reduced susceptibility to treatment and increased autophagy activation. Thus, modulating autophagy may be a potential strategy to enhance PDT efficacy in melanoma [26].

Highlights:

- Cytotoxicity, microvascular killing, and immune response activation are the basic therapeutic PDT induced mechanisms in skin cancer.
- Apoptosis, necrosis, ferroptosis, and ICD are the main PDT-induced cell death pathways in skin cancer.
- Apoptosis and necrosis would appear to be dependent on the grade of OS.
- In melanoma, PDT-induced autophagy would be recognized as having a presumed cytoprotective role.

## 4. Pancreatic Cancer

Pancreatic ductal adenocarcinoma (PDAC) is predicted to become the second leading cause of cancer-related deaths in the United States by 2030. This grim prognosis is primarily due to late-stage diagnosis, occurring in approximately 90% of cases, and as a result metastatic spread, which is observed in over 50% of patients. In this critical epidemiological context, there is an urgent need for early diagnostic strategies and effective therapies to target and eliminate metastatic PDAC cells [27].

In clinical settings, PDT shows promise for pancreatic cancer, particularly for palliative care in advanced stages or as an adjuvant therapy. It can be used preoperatively to downstage the tumor or to address micrometastatic lesions, aiding in the achievement of an R0 resection post-operatively [11]. Mechanistically, the efficacy of PDT in pancreatic cancer relies on its ability to induce necrosis and apoptosis of cancer cells, damage tumor vasculature, and stimulate antitumor immunity. Specifically, integrin-targeted PDT approaches have been shown to cause S-phase cell cycle retardance and inhibit cell proliferation, ultimately leading to apoptosis induction. Moreover, maltotriose-chlorin has been identified as the first PS capable of inhibiting ascites formation by inducing irreversible apoptosis in peritoneal disseminated tumor cells. Additionally, verteporfin-mediated PDT is employed in this malignancy due to its ability to trigger apoptosis through the oxidation of key molecules involved in cell proliferation [11]. Beyond its pro-oxidant potential, verteporfin has recently been discovered to induce ferroptosis independently of light activation in pancreatic cancer cells [28]. The rationale for using verteporfin as a PS in this malignancy is also justified by its role as a small molecular inhibitor of the Hippo-Yes-associated protein-1 (YAP) pathway, which is critically involved in the proliferation of human PDAC cells. Mechanistically, this inhibition occurs by disrupting the interaction between YAP and TEAD, leading to suppressed expression of their target genes. Additionally, verteporfin exerts inhibitory effects on angiogenesis and vasculogenic mimicry by suppressing the expression of Angiopoietin-2 (Ang2), matrix metalloproteinase (MMP)-2, vascular endothelial (VE)-cadherin, and α-Smooth Muscle Actin (SMA), both in vitro and in vivo [29].

In addition to apoptosis, MB-PDT-induced necroptosis has recently emerged as a novel cell death mechanism in human PDAC cells. This process is associated with enhanced cytotoxicity, potentially opening new clinical applications for this approach in the treatment of microscopic residual disease and in minimizing the likelihood of local and metastatic recurrence [30].

Even more intriguingly, an emerging area of research focuses on PDT’s ability to impact the fibrotic stroma of PDAC, which is notoriously a barrier to drug delivery and a critical hub for pro-tumorigenic signaling. Preliminary evidence suggests that PDT may play a role in depleting both cellular (fibroblasts, pancreatic stellate cells) and non-cellular (ECM breakdown) stromal components of PDAC. This disruption could interfere with the stromal signaling crosstalk, essential for tumor survival [31,32,33].

Groundbreaking and highly promising are also the initial in vivo studies on murine models of PDAC, which explore the ability of fibroblast activation protein (FAP)-targeted PDT to induce localized depletion of FAP-expressing stromal cells, thereby exerting an even more significant impact on the tumor-supporting stroma [34,35].

Concerning the stimulation of anti-cancer immunity recent studies have highlighted PDT’s ability to induce pyroptosis, a highly inflammatory form of ICD, in pancreatic cancer. This process promotes M1 macrophage polarization, dendritic cell (DC) maturation, and cytotoxic T lymphocyte (CTL) activation as hallmarks of a systemic immune response. These effects, alongside regulatory T cell (Treg) suppression, facilitate the conversion of the tumor microenvironment from “cold” (immunosuppressive) to “hot” (immunogenic), thereby inhibiting both primary tumor growth and distant metastasis [36,37]. On the other hand, the pro-metastatic potential associated with low (sublethal) doses of PDT in peripheral pancreatic cancer cells cannot be overlooked. This effect is thought to occur through the upregulation of MMP-2/9 expression, as well as the enhanced motility and invasion of residual pancreatic cancer cells [38].

Highlights:

- Direct cytotoxicity, damage of tumor vasculature, and stimulation of antitumor immunity are the three mechanisms underlying the efficacy of PDT in pancreatic cancer.
- Apoptosis, necrosis, pyroptosis, and ferroptosis (independent of light activation) are the main PDT-induced cell death pathways in pancreatic cancer.
- Promising role of Verteporfin-PDT through its anti-angiogenic effects and inhibition of Hippo-YAP pathway involved in PDAC cell proliferation.
- The ability of FAP-targeted PDT to impact the dense tumor stroma represents an innovative and promising line of research.

## 5. Cholangiocarcinoma (CCA)

CCA is a rare neoplasm with an increasing incidence, characterized by insidious clinical progression and challenging diagnosis. Palliative treatments, including biliary drainage, chemotherapy, radiotherapy, targeted therapy, and immunotherapy, are the primary approaches for managing CCA. However, these treatments often offer limited improvements in quality of life and survival [39,40]. Preliminary clinical studies suggest that PDT may be beneficial for palliative care in CCA. Additional clinical advantages include a reduction in biliary ductal stenosis in unresectable cases, as well as improved quality of life and extended survival without significant additional adverse events. These findings were supported by a recent meta-analysis focused on hilar CCA [41].

The clinical application potential of PDT is highly promising, particularly in gemcitabine-resistant CCA. At the molecular level, recent studies have highlighted the effect of PDT on the Krueppel-like factor 10 (KLF10)/epidermal growth factor receptor (EGFR) axis. Specifically, PDT promotes KLF10 expression, which binds to the EGFR promoter region, thereby inhibiting EGFR transcription. This results in suppressed cell viability, G1 cell cycle arrest, and the induction of cellular apoptosis [42].

The induction of apoptosis also involves epigenetic mechanisms, with a critical role played by miR-34a-5p. In CCA cells, PDT-induced p53 upregulation has been shown to mediate the transactivation of miR-34a-5p, which subsequently downregulates Wnt Family Member 7B (WNT7B) expression. This, in turn, inhibits Wnt signaling, a pathway associated with cell proliferation [43].

Even more interestingly, Yan et al., in a recent in vivo and in vitro study, demonstrated the ability of HiPorfin-mediated PDT to trigger multiple mechanisms of cell death in CCA cell lines. In addition to the well-known apoptosis, ferroptosis has emerged as a complementary cell death pathway, which appears to be associated with enhanced therapeutic efficacy. This was in vivo evidenced by a reduction in tumor tissue and malignancy grade. The underlying molecular mechanisms involve the activation of the P53/Solute Carrier Family 7 Member 11 (SLC7A11)/Glutathione (GSH) peroxidase 4 (GPX4) ferroptosis signaling pathway through the inhibition of GSH synthesis-related pathways [44]. Intriguingly, in CCA cells, Chlorin A-PDT has also been shown to induce autophagy as an additional cell death mechanism essential for triggering apoptosis. At the molecular level, the activation of the ER stress-related Protein Kinase RNA-Like ER Kinase (PERK)/Phosphorylation of the eukaryotic initiation factor 2 (p-EIF)2α/CCAAT-enhancer-binding protein homologous protein (CHOP) pathway plays a crucial role in initiating these processes [45]. In addition to its previously discussed antitumor effects, the PDT-induced extensive activation of survival pathways in CCA cells should not be overlooked, especially in sublethally afflicted tumor cells. These include activator protein 1 (AP-1), NF-κB, hypoxia-inducible factor 1-alpha (HIF-1α), nuclear factor erythroid 2-related factor 2 (NFE2L2), and pathways mediated by the unfolded protein response (UPR), responsible for a suboptimal therapeutic response. Indeed, all these pathways are essentially involved in the transcriptional regulation of pro-tumoral cellular events, including proliferation, energy metabolism, detoxification, inflammation/angiogenesis, and metastasis [46,47].

Highlights:

- Apoptosis, ferroptosis, and autophagy are the main PDT-induced cell death pathways in CCA.
- In gemcitabine-resistant CCA, apoptosis is mainly related to the PDT-induced KLF10/EGFR axis.
- In CCA cells, apoptosis is also epigenetically PDT-induced via Wnt signaling inhibition.

## 6. Hepatocellular Carcinoma (HCC)

HCC is a significant global health issue, especially in the context of the obesity epidemic, which has made non-alcoholic fatty liver disease a major risk factor. According to the latest epidemiological data, HCC represents the sixth most common malignancy and the fourth leading cause of cancer-associated mortality in the world. However, prognosis has been improving over the years, thanks to the development of more effective systemic and locoregional therapies [48]. In this scenario, there is justified optimism regarding the therapeutic prospects offered by PDT, especially considering its well-known immunoregulatory role, which suggests its potential use in the adjuvant setting. Specifically, the intra-operative administration of PS and the fluorescence generated from their excitation enhance visual detection, allowing the surgeon to identify subclinical nodules and guide optimal liver resection. Following this, PDT can target any residual lesions and activate an antitumor immune response [49]. Given this context, the growing interest in understanding the underlying mechanisms behind this promising new therapeutic frontier is well-justified. Assuming that the escape of cancer cells from apoptosis is a major driver of their uncontrolled proliferation, the antiproliferative effects of PDT, which can trigger pro-apoptotic pathways, has been widely investigated. Radachlorin-based PDT has been shown to primarily induce apoptosis in human liver cancer HepG2 cells [50]. In the same cell line, ALA-based PDT has also been demonstrated to induce deoxyribonucleic acid (DNA) damage, leading to cell death via apoptosis and/or necrosis [51]. Further evidence supports that necrosis is a significant PDT-induced cell death mechanism alongside apoptosis [52].

Molecularly, the upstream target of this PDT tumor-killing activity appears to be the suppression of the proliferative MAPK-signaling pathway [53].

Moreover, a recent notion is that the diverse molecular and infectious characteristics of HCC tumor cells influence their PDT-induced pro-apoptotic response. In studies using 5-ALA-based PDT on HCC cell lines Huh-7 and SNU-449, an antiproliferative effect and subsequent induction of apoptosis were observed, with SNU-449 cells showing significantly higher sensitivity compared to Huh-7 cells. This differential response appears to depend on the hepatitis B virus status and specific molecular characteristics of the cells, such as point mutation in the p53 gene in Huh-7 cells and a deletion of the cyclin dependent kinase (CDK) inhibitor 2A gene in SNU-449 cells [54].

Concerning the recently emerged anti-inflammatory potential, it is important to note that the inflammatory tumor microenvironment (TME) plays a significant role in various stages of HCC development, with NF-κB acting as a crucial proinflammatory modulator through its transcriptional activation. In this inflammatory setting, neutrophils are activated, migrate, and accumulate in the tumor tissue. Building on these pathophysiological insights, the use of neutrophil membrane-Hypocrellin B nanoparticle-mediated PDT represents an interesting development in HCC treatment. This approach has shown a significant reduction in expression levels of TNF-α and IL-6, indicating a systemic anti-inflammatory effect. Additionally, it induces mitochondrial ROS generation and depolarization in HepG2 cells, via inhibited junB expression and subsequent apoptosis [55].

Last but not least, it is well established that most anti-cancer therapies cause a reorganization of the cellular immune landscape within the TME, either promoting or inhibiting tumor growth. An ideal anti-cancer therapy should not only have destructive effects on the tumor but also enhance the immune system against the neoplasm itself. In this view, the immunostimulatory ability of PDT in HCC is significant and warrants further investigation. Recent in vivo studies have reviewed the effects of deuteporfin-mediated PDT on mice hepatomas, demonstrating its ability to induce maturation and activation of DCs, which in turn can significantly reduce tumor growth. This immunogenic potential is further supported by evidence showing that pheophorbide (Pheo)-mediated PDT induces ICD by activating macrophage phagocytosis of cancer cells. Consequently, this leads to the processing and presentation of tumor-associated antigens, stimulating an antigen-specific T cell response. PDT also promotes the clonal expansion of human peripheral blood mononuclear cells, further supporting its role in enhancing immune responses against cancer [49,56].

Highlights:

- Apoptosis and necrosis may both be major mechanisms of PDT-induced cell death in HCC.
- PDT-induced ICD is part of a more extensive antitumor immunostimulatory role of PDT in HCC.
- PDT-modulated cell death-related pathways: apoptosis—MAPK-signaling pathway;

ICD—macrophage phagocytosis/processing and exposure of tumor-associated antigens/antigen-specific T cell response.

## 7. Gastric Cancer (GC)

GC is the fifth most common cancer and the third leading cause of cancer-related death worldwide. Due to its phenotypic and molecular variability, it is considered a highly heterogeneous disease [57]. Recent epidemiological revisions indicate that this heterogeneity extends to its geographical distribution. East Asia has the highest incidence rates, followed by Eastern and Central Europe [58]. In the therapeutic setting, PDT is emerging as a promising “light knife” approach for treating this neoplasm at various clinical stages, thanks to its non-invasive nature and minimal side effects. Encouraging clinical results have been observed with the use of PDT in treating early gastric cancer, as well as in its application as an adjunct treatment during endoscopy. In cases of inoperable advanced gastric cancer, particularly when associated with peritoneal metastases, as well as cardiac and pyloric obstructions, PDT may prove valuable for palliative care, thereby improving the patient’s quality of life [59]. Current research trends focus on elucidating the cell death pathways engaged and the role of PDT in stimulating antitumor immunity.

Specifically, in various GC cell lines, several PSs-mediated PDT approaches have been observed to induce a dose-and energy-dependent antiproliferative effect. This is achieved through cell cycle arrest in the S-phase and subsequent induction of apoptotic cell death via the mitochondrial pathway, as indicated by the upregulation of caspase-9 and caspase-3 protein levels [60,61,62].

Additionally, apoptosis appears to be mediated not only by mitochondrial mechanisms but also by ER stress. It is known that cellular stress related to the accumulation of unfolded or misfolded proteins at the ER level is a powerful driver for the onset of various chronic diseases, including cancer. In this context, the UPR is the signaling pathway deputed to ensure surveillance of ER proteostasis. However, chronic cell damage can lead this pathway to induce apoptotic cell death [63]. This pathophysiological rationale is exploited by PDT in cancer. In the human GC cell line MKN45, 2-(1 hexyloxyethyl)-2-devinyl porphin e6 trisodium salt (HCE6)-mediated PDT induces mitochondria and ER-mediated apoptosis. Specifically, the mitochondrial pathway is activated via upregulation of proapoptotic ankyrin repeat domain 1 and downstream release of cyt-c. Concurrently, ER-mediated apoptosis is triggered via upregulation of the glucagon receptor and junctional sarcoplasmic reticulum protein 1, along with increased levels of HSP70, HSPA6, HSPA7, and antisense ribonucleic acid (RNA) IDI2-AS1 [64].

In addition to apoptosis, necrosis and autophagy have recently emerged as additional PDT-induced cell death pathways in RGK1 gastric cancer-like cells [65].

As previously mentioned, a promising PDT role in stimulating antitumor immunity is being actively investigated for GC. This is particularly relevant in the era of immunotherapy, as advancements in this area could enhance the clinical management of this malignancy by synergizing with existing immune checkpoint inhibitors. Such progress could improve therapeutic efficacy by converting “cold tumors” into “hot tumors”, characterized by increased cellular immune infiltration. PDT’s role in promoting intratumoral immune cell infiltration is noteworthy, as it fosters specific clonal expansion of CTLs while reducing the presence of Tregs. Moreover, PDT enhances interactions between tumor cells and effector cells, while disfavoring interactions between Tregs and other immune cells. In this context, PDT not only promotes co-stimulatory signaling but also disrupts co-inhibitory signaling [66].

Highlights:

- Apoptosis, both mitochondria-and ER-mediated, appears to be the main mechanism of PDT-induced cell death in GC. However, there is recent evidence of alternative pathways which have been identified in autophagy and necrosis.
- PDT-induced cell death-related pathways: intrinsic mitochondrial apoptosis—ankyrin repeat domain 1/Cyt-c/caspase-9/caspase-3; ER-mediated apoptosis—glucagon receptor/junctional sarcoplasmic reticulum protein 1/HSPs.
- The emerging antitumor immunity-stimulating role of PDT in GC relies on CTLs clonal expansion and simultaneous Treg restriction.

## 8. Esophageal Cancer

Esophageal cancer is the sixth most common cause of cancer-related death worldwide, with esophageal squamous cell carcinoma (ESCC) being the predominant histotype. Over the last decade, new treatment strategies have emerged and are replacing traditional approaches in all stages of this neoplasm [67]. In this era of announced therapeutic revolution, PDT is already an approved endoscopic ablative practice for this neoplasm in some countries. It is also approved for dysplastic Barrett’s esophagus and is used for palliative purposes in symptomatic obstructive esophageal cancer. A further impetus for its use comes from diminished phototoxicity thanks to the use of second-generation PSs [68]. In this context, there is a growing body of preliminary evidence aimed at elucidating the cytomolecular mechanisms underlying its therapeutic efficacy. Research is focusing on three main areas: the effects of PDT on the induction of cell death mechanisms and its anti-migratory properties, the stimulation of antitumor immunity, and the suppression of cancerous aerobic glycolysis (also known as the Warburg effect) [68].

Regarding its immunoregulatory role, evidence suggests that PDT has a stimulatory potential on both innate and acquired host immunity. In a clinical setting, these cellular events lead to tumor regression and inhibited tumor recurrence, contributing to the improved long-term survival rates reported after PDT. A significant aspect of this process involves the modulation of immune cells which are crucial in maintaining immune homeostasis. In patients with invasive ESCC, PDT has been shown to inhibit the immunosuppressive functions of peripheral cluster of differentiation (CD)4 + CD25 + CD127-forkhead box P3 (foxp3) + Tregs. Consequently, there is a substantial increase in circulating neutrophilic granulocytes and monocytes [69].

Beyond this immunomodulatory role, PDT’s applicative potential in esophageal cancer also includes its direct antitumor action on cancer cells, which is further enhanced by the use of novel PSs designed to minimize side effects on surrounding healthy tissues. Hu et al. revealed the in vitro antiproliferative efficacy of Sinoporphyrin sodium (DVDMS)-mediated PDT on human esophageal cancer Eca-109 cells. Their study points out a significant induction of ROS production, leading to reduced cell viability, DNA damage, and loss of mitochondrial membrane potential [70].

Further molecular findings in Eca-109 cells concern the ability of DVDMS-mediated PDT in inducing a ROS-dependent activation of p38 MAPK, JNK, and eme-oxygenase 1 (HO-1) upregulation. This activation is responsible for inducing apoptosis, which is also functionally supported by the induction of autophagy [71].

However, the induction of the apoptotic process is not limited to the above-mentioned molecular involvement but also involves NF-κB. This factor is known to be a key modulator of various cellular processes such as proliferation, apoptosis, immunity, angiogenesis, and embryonic development. Upon activation by various stimuli, including cytokines, OS, DNA damage, infection, or others, NF-κB is transported from the cytoplasm to the nucleus, where it triggers the transcription of target genes. In human esophageal cancer cell lines Eca109 and Ec9706, dihydroartemisinin (DHA)-mediated PDT has been shown to inactivate ROS-induced NF-κB activity. This results in significant downstream effects, including the downregulation of the anti-apoptotic Bcl-2 protein, upregulation of Bax, and increased activation of caspase-3 and caspase-9 [72]. Specifically, the DHA-PDT antitumor role was also revealed in the suppressing ability of NF-κB/HIF-1α/vascular endothelial growth factor (VEGF) pathway [73].

Furthermore, there is substantial evidence of EGFR overexpression in esophageal cancer, leading to the upregulation of its two downstream PI3K/AKT and Ras/Raf/MAPK signaling pathways. Both pathways play significant roles in the malignant biological behavior of cancer cells, including proliferation, apoptosis, angiogenesis, invasion, and metastasis. In ALA-PDT treated esophageal carcinoma cells Eca-109, the governing antitumoral mechanism was identified as the downregulation of certain molecules within the EGFR/PI3K/AKT signaling pathway [74].

The involvement of the PI3K/AKT pathway in other cancer cell functions includes a key role in cell migration, which is associated with epithelial–mesenchymal transition (EMT). A crucial marker of this event is the so-called “cadherin switch”, characterized by downregulation of E-cadherin and concomitant N-cadherin up-regulation. This results in reduced epithelial intercellular adhesion and subsequent promoted cell migration. In ESCC cells, hematoporphyrin derivative-mediated PDT showed significant ROS-generating potential, associated with a reduction in cell viability and migration. This effect is achieved by inhibiting the EMT process via the upregulation of E-cadherin and downregulation of N-cadherin. The anti-migratory effect was further supported by the induction of cell apoptosis and autophagy. The molecular mechanism governing these cellular phenomena is identified in the PDT-mediated downregulation of the PI3K/AKT/mTOR signaling pathway [75].

A further field of research worthy of attention is the influence of PDT on aerobic glycolysis in cancer cells. This metabolic process, characterized by a dramatic increase in glycolysis even in the presence of oxygen, is a hallmark of cancer metabolism and a promising therapeutic target. Known as the Warburg effect, this phenomenon reflects an adaptive mechanism that cancer cells use to meet the demands of uncontrolled proliferation. Despite having functional mitochondria and the presence of oxygen, cancer cells exhibit increased glucose uptake and lactate production. Gan et al. evaluated the influence of 5-ALA-PDT on the Warburg effect in esophageal cancer cells. Their study highlighted that 5-ALA-PDT downregulates pyruvate kinase M2 (PKM2), a key speed-limiting enzyme of cancer cell glycolysis, together with inhibition of glucose uptake and lactate production. This initial (after 4 h) antiglycolytic effect was followed, at a late post-PDT period (after 24 h), by significant PKM2 upregulation, thus highlighting the time-dependent regulatory role of ALA-PDT on cancer cell glycolysis [76].

Furthermore, closely related to PKM2 downregulation is the induction of pyroptosis as a novel mechanism of cell death. In Eca109 and Ec9706 cancer cell lines, PDT has been shown to downregulate PKM2, resulting in the activation of caspase-8 and caspase-3, and downstream release of N-gasdermin E (GSDME) activating pyroptosis. This is revealed by an increase in proinflammatory factors TNF-α, IL-1α, IL-1β, IL-6, and interferon (IFN)-γ) [77].

In addition to pyroptosis, necrosis and ferroptosis also represent key cell death pathways induced by PDT in esophageal cancer cells. Recent studies have highlighted the role of ALA-PDT in inducing various cell death pathways, including apoptosis, necrosis, and ferroptosis, in esophageal Kyse 450 carcinoma cells. Notably, ALA-PDT was observed to alter local extracellular levels of DAMPs. Specifically, there was an increase in adenosine triphosphate (ATP) amounts and a decrease in HMGB1, alongside an increase in extracellular lipid peroxidation products. These changes may influence macrophage phenotype and potentially activate antitumor immunity, highlighting an area of exploration for future research [78].

Highlights:

- Apoptosis (also functionally supported by autophagy) is configured as the main mechanism of PDT-induced cell death in esophageal cancer cells. On the other hand, PDT-induced PKM2 downregulation is responsible for GSDME-mediated pyroptosis.
- PDT-influenced cell death-related pathways:
  Apoptosis: NF-κB/HIF-1α/VEGF and PI3K/AKT/mTOR signaling.
  Pyroptosis: PKM2/caspase-8/caspase-3/GSDME.
- There is emerging evidence on the antitumor immunoregulatory role of PDT via inhibition of Tregs’ suppressive functions as well as by supposed alterations in extracellular levels of DAMPs.
- An antiglycolytic (even time-dependent) role of various PDT approaches is emerging. This anti-Warburg effect is molecularly founded on PKM2 downregulation.

## 9. Colorectal Cancer (CRC)

CRC is the third most common cancer and the second leading cause of cancer-related death in the world [79]. Beyond its classical definition applicable to late-onset CRC, clinical interest has been progressively increasing in recent decades due to the increased incidence rates of early-onset CRC (<50 years old). The use of PDT for CRC shows promise, particularly as a new palliative treatment for advanced cases. However, significant challenges remain, including issues related to PDT resistance [80,81,82]. In this clinical-epidemiological setting, the importance resulting from an increased cytomolecular understanding of PDT’s antitumor potential is crucial. The three main research strands focus on (1) PDT-induced cell death phenomena, (2) the role of PDT in cytoskeletal reorganization for antimigratory purposes, and (3) PDT’s stimulatory action on antitumor immunity.

The involvement of MAPKs on elicited cell death pathways is essential. MAPKs are activated in response to various cellular stimuli and are essentially involved in the regulation of cellular processes, including apoptosis and autophagy. Among MAPKs, p38 MAPK is specifically activated by stress stimuli like OS, ultraviolet irradiation, or hyperthermia, leading to apoptosis [83]. This underscores the importance of p38 MAPK as a key molecular target for PDT in cancer therapy.

Xue et al. studied the in vitro effects of chlorin e6 (Ce6)-mediated PDT in human colorectal cancer SW620 cells. Its ability for ROS generation and downstream p38 MAPK activation was identified, leading to the induction of apoptosis and autophagy. Notably, autophagy may represent a cytoprotective mechanism, as p38 MAPK activation appears to mitigate Ce6-PDT-induced cell apoptosis [84].

The regulatory role of p38 MAPK was also investigated in tetra-α-(4-carboxyphenoxy) phthalocyanine zinc (TαPcZn)-PDT-in LoVo human colon carcinoma cells. The study revealed a key involvement of p38 MAPK in the crosstalk with caspase-9 during TαPcZn-PDT-induced mitochondria-mediated apoptosis [83].

Interestingly, PDT’s molecular targeting can extend to the epigenetic level, thanks to advances in the discovery of an increasing number of CRC-related non-coding RNAs and miRNAs. Among these, miR-124 is well known for its tumor suppressive role on CRC, as well as for its interaction with various oncogenic transcripts, including Nuclear Enriched Abundant Transcript 1 (NEAT1). Recent studies have shown that 5-ALA-PDT induces apoptosis in colorectal cancer p53 wild-type HCT116 and RKO cells. This effect includes the downregulation of NEAT1 (via the downregulation of c-myc) and the upregulation of miR-124 expression, which is associated with a decrease in proliferating cell nuclear antigen levels [81].

In addition to apoptosis, a newly emerged PDT-induced cell death pathway has been identified in pyroptosis. In CT26 and HCT116 cells, mitochondria-targeted PDT induces pyroptosis via ROS formation and the promotion of downstream p38 phosphorylation. This process leads to the active caspase 3 (CASP3)-mediated cleavage of GSDME within the ROS/p38/CASP3/GSDME axis, thereby inhibiting CRC progression [85]

The cytoskeleton is a crucial target in anti-cancer therapy, as are all signaling molecules and processes that contribute to cancer cell migration and metastasis. The cytoskeleton is specifically supported by the combined action of pseudopodia, cytoskeletal aggregation, and depolymerization. Key regulatory events involved in cytoskeletal reorganization and cell migration are controlled by Ras homology (Rho) family proteins, of which Ras-related C3 botulinum toxin substrate 1 (Rac1) is a highly expressed member in many CRC cells and plays a key role in cell migration [82].

Wufuer et al. evaluated the in vitro anti-migratory effects of Ce6-PDT on SW620 human CRCs. They identified the downregulation of the Rac1/p21 (Rac1) activated kinase 1/LIM domain kinase 1/cofilin signaling pathway as a key factor in the depolymerizing ability of the microfilament structure. This was combined with a significant reduction in F-actin expression, in turn responsible for the decrease or disappearance of cell pseudopodia [82]. These cytoskeletal events were further corroborated by observations of a marked decrease in F-actin, α-tubulin, β-tubulin, and vimentin, along with an increase in E-cadherin. All these mechanisms were found to be responsible for the inhibition of proliferation and migration of SW480 cells following Ce6-PDT [86]. Building on preliminary evidence, the effects of hypericin (Hyp)-mediated PDT on metastatic CRC cells have recently been evaluated in vitro. It has been revealed Hyp-PDT’s anti-invasive and anti-migratory ability, evidenced by decreased F-actin formation, destruction of pseudopodia formation, upregulation of E-cadherin, and downregulation of N-cadherin and vimentin. Additionally, Hyp-PDT suppressed EMT. These events are linked to the downregulation of RhoA/Rho-associated, coiled-coil-containing protein kinase 1 signaling pathway in metastatic colorectal HCT116 and SW620 cells [87].

Furthermore, research into the antitumor immunostimulatory potential of PDT is gaining traction, especially in the context of immunotherapy. This is particularly relevant given the potential synergy between PDT and immune checkpoint inhibitors, which have already shown success in treating various malignancies, including CRC. In a mouse model of CRC, Ce6-PDT was found to increase CD8 + activated T cells, CD8 + CTLs, and CD8 + naïve T cells while decreasing exhausted CD8 + T cells and CD4 + effector memory T cells. PDT also promoted CD8 + T cell infiltration and their functional activation in TME, evidenced by increased release of key effector molecules, including granzyme B (Gzmb) and perforin 1. Notably, this antitumor immune activation appears to be systemic, as indicated by elevated serum levels of Gzmb, IFN-γ, and IL-2 [88].

Highlights:

- Apoptosis, besides the newly emerged pyroptosis, is the main mechanism of PDT-induced cell death in CRC, while PDT-induced autophagy performs a tumor cytoprotective role.
- PDT-influenced cell death-related pathways:
  Mitochondrial apoptosis: p38 MAPK/caspase-9/caspase-3.
  Pyroptosis: ROS/p38/CASP3/GSDME.
- There is growing evidence for a PDT-reorganizing role of cytoskeletal architecture for anti-migratory purposes in CRC cells.
- There is encouraging evidence of an antitumor immunostimulatory role for PDT, primarily involving CD8 + T cells.

## 10. Osteosarcoma (OS)

OS is the most common primary bone malignancy. Its incidence is now characterized by a unimodal distribution, with a significant peak occurring in the second decade of life [89]. Over the past decade, there has been a growing interest in systematically re-evaluating data to better understand the effectiveness and safety of PDT for this neoplasm. To date, there are no clinical reports demonstrating the curative potential of PDT for human OS. However, it could potentially serve primarily as a palliative treatment [90]. A thorough evaluation of the signaling pathways being PDT modulated has been lacking, with preliminary evidence only addressing molecular pathways that either promote or inhibit various mechanisms of cell death.

Among second-generation PSs, hiporfin-mediated PDT has been widely investigated. In OS, the induction of various cell death pathways differs depending on the cell line under investigation. Apoptosis has been observed in DLM-8, 143B and HOS cells; necroptosis has been observed in DLM-8 cells; and autophagy has been observed in 143B and HOS cells. Molecularly, apoptosis is indicated by increased levels of cleaved-caspase-3, cleaved PARP-1 and Bax, along with reduced expression of Bcl-2. Increased levels of receptor-interacting protein1 indicate necroptosis, while LC3I to LC3II conversion is a marker for autophagy, which is believed to have a protective role [91].

Both apoptosis and autophagy are pathways induced by ROS-JNK pathway activation. Janus signaling exhibits a “Yin and Yang” effect, which is particularly evident in PDT treated OS. JNK plays a mediating role, functioning both as a pro-apoptotic agent and as a mediator of cancer cell survival via an autophagic process. Moreover, this pro-autophagic effect has been associated with the development of chemotherapeutic resistance [92].

In the human OS cell line MG-63 treated with AE-PDT, there was a notable overexpression of both the apoptotic protein cleaved caspase-3 and the autophagic proteins LC-3II and Beclin-1. This molecular change is attributed to a time-dependent p-JNK overexpression, indicating essential ROS-JNK pathway involvement. However, a presumed autophagic protective mechanism occurs only in the early stage of therapy [93]. A similar study on the effects of Pheo-α methyl ester (MPPa)-mediated PDT on human OS MG-63 cells evidenced comparable ROS-JNK pathway activation ability, leading to both autophagic and mitochondrial apoptotic responses [94].

Additionally, the ROS-JNK pathway is not the only survival pathway being PDT-modulated. In HOS cells treated with MPPα-PDT, a further resistance mechanism recognizes the crucial involvement of p21, an effector of the PERK-activating transcription Factor 4 (Atf4) pathway [95].

Beyond the dichotomous role of the ROS-JNK pathway, a clear pro-survival role involving the PI3K/AKT/mTOR pathway highlights this pathway as a potential PDT target. In the human OS cell line MG-63, MPPa-PDT inhibits proteins in the PI3K/AKT/mTOR pathway, further contributing to the suppression of tumor cell proliferation, angiogenesis, and metastasis. Specifically, its antiproliferative action involves arresting the cell cycle in the G2M phase, combined with ER stress-induced apoptosis. Additionally, MPPa-PDT-induced antitumor effects are enhanced by inhibiting cell invasion and migration via the downregulation of MMP-2 and MMP-9 [96].

However, the functional dualism of PDT involves various other molecular actors, with a close association observed between the promotion of antioxidant signaling and cancer cell survival mechanisms.

Zhan et al. provided additional evidence supporting a pro-apoptotic role of MPPa-PDT in HOS cells. Nevertheless, the MPPa-PDT-mediated induction of YAP represents an emerging and significant mechanism of therapeutic resistance. YAP’s role in suppressing apoptosis contributes to enhanced cancer cell proliferation [97]. Additionally, in human HOS cells, MPPα-PDT also activates X-box binding protein 1 (XBP1), a downstream product of the inositol-requiring enzyme 1 α (IRE1α)-XBP1 pathway, which is a key mediator of resistance mechanism to PDT. This activation occurs via MPPα-PDT-induced upregulation of antioxidant-related proteins, such as catalase (CAT) and superoxide dismutase (SOD)-1 [98].

Besides the conflicting PDT-induced molecular functions, a functional synergism between nanoparticle-guided PDT induced apoptosis and ferroptosis has also been observed in HOS cells. This antitumor synergism is elicited by PDT-induced inactivation of glutathione peroxidase 4, alongside the activation of nuclear receptor coactivator 4-mediated ferritinophagy [99].

Highlights:

- In OS, hiporfin-mediated PDT induces various cell death pathways depending on the cell lines used.
- Autophagy is a presumed tumor protective mechanism against PDT.
- PDT-influenced cell death-related pathways.
  ROS-JNK pathway activation by various PDT approaches is crucial for the downstream activation of apoptotic and autophagic pathways.
- In OS, the activation of PI3K/AKT/mTOR and ROS-JNK pathways is crucially induced by MPPa-PDT for antitumor purposes. Conversely, MPPα-PDT-induced activation of IRE1α-XBP1 and PERK-Atf4 pathways is a mechanism of tumor resistance.

## 11. Blood Cancer

In the oncohematological field, particularly for adult T-cell leukemia/lymphoma (ATL), PDT represents a promising new approach due to its restricted side effects compared to radiotherapy and chemotherapy. The current era is moving towards clinical applications for PDT in this field. Previous reviews have mainly highlighted the efficacy of ALA-PDT in eliminating ATL leukemic cells by inducing leukemic cell death via apoptosis and/or necrosis. Clinically, this would lead to the inhibition of ATL progression from an indolent to an aggressive form [100].

Given these promising prospects, the last decade has witnessed a growing international scientific interest in elucidating both the underlying antitumor and pro-tumor mechanisms induced by PDT in lymphoma- and leukemia-derived cell lines.

Among the various PSs investigated, significant scientific attention has been directed toward the excellent photochemical properties of hypericin. This plant-derived compound has demonstrated selective accumulation in tumor tissue through mechanism such as diffusion, endocytosis, or pinocytosis. Its efficacy as a PS is attributed to its ability to generate high singlet oxygen. In Jurkat cells, Hyp-PDT pro-apoptotic effects have been linked to the activation of the TNF-related apoptosis-inducing ligand (TRAIL)/TRAIL-receptor system and caspase-8, while in ATL cells, it induces mitochondrial apoptotic activation. In U937 cells, Hyp-PDT led to reduced cell viability and apoptosis, as evidenced by altered levels of Bcl-2, Bax, and PARP. Furthermore, in a P388 murine lymphoma model, Hyp-PDT induced tumor vasculature damage [101].

Among the several pathophysiological pathways regulating apoptosis, the JNK pathway plays an essential role. JNK, activated by various cellular stressors can act in a bimodally fashion, either promoting or inhibiting apoptosis depending on the type of stressor event and the specific cell type involved. In K562 leukemia cells, Hyp-PDT triggers mitochondria-caspase-dependent apoptosis via the upstream activation of P-JNK and subsequent upregulation of cleaved caspase-9 and cleaved caspase-3 [102]. Further support for this apoptotic mechanism was provided by studies on phenalenone-induced PDT in the human tumor cell line HL60 (acute promyelocytic leukemia). Notably, ROS generation via both type I and type II mechanisms induced both extrinsic (direct activation of caspase-3) and intrinsic (mitochondrial depolarization) apoptotic pathways, initiated by upstream activation of caspase-8/tBid and p38-MAPK. This process showed a clear predominance of apoptosis over resistance pathways mediated by PI3K/AKT and JNK, simultaneously activated by PDT [103].

Based on the reviewed evidence, mitochondrial apoptotic activation remains the primary mechanism of PDT-induced cell death in the oncohematologic setting. This was further supported by findings in the rat acute myeloid leukemia cell line LT12, where PsD007-mediated PDT led to the upregulation of cleaved caspase-7, caspase-9, PARP, and cleaved caspase-3 [104].

Nevertheless, evidence for PDT-induced caspase-independent apoptosis is limited, despite a broader activation of non-apoptotic cell death pathways. Specifically, PDT-induced lysosomal ROS generation can lead to the cytoplasmic release of cathepsins, which are crucial for the initiation of various cell death pathways. For instance, in mouse leukemia cells L1210 treated with the novel Acridin-3,6-dialkyldithiourea hydrochlorides-mediated PDT, ROS formation and cell cycle arrest in the subG0 phase were observed, as along with induction of multiple cell death types, such as autophagy, caspase-independent apoptosis, and/or necrosis [105].

Highlights:

- Lymphoma and leukemia are the hematological malignancies mainly investigated concerning molecular and cellular pro/antitumor mechanisms elicited by PDT.
- Various PDT approaches appear to elicit apoptosis, both caspase-dependent and caspase-independent, as the predominant mechanism of cell death. Minor evidence of autophagy and necrosis as alternative/complementary pathways of cell death is also evidenced.
- PDT-influenced cell death-related pathways:
  Apoptosis: TRAIL/TRAIL-receptor system/caspase-8, intrinsic (P-JNK/caspase-9/caspase-3) and extrinsic (caspase-8/tBid/caspase-3).
- There is preliminary evidence for the prevalence of the apoptotic pathway over the resistance pathway, equally and simultaneously activated by PDT.

## 12. Prostate Cancer

Prostate cancer is the leading cause of cancer mortality in Western countries, albeit with epidemiological differences observed between countries, caused by the interplay of multiple influencing factors, such as genetic, environmental and social factors. Moreover, each canonical treatment available currently is associated with severe side effects, along with the development of resistance to the initial treatment. This explains the need to move towards new therapeutic strategies that are both effective and inexpensive and well-tolerated. Nanomedicine, gene therapy and phytotherapy offer promising perspectives in this regard [106]. The PDT shows promise as a focal therapy for men with localized low-and intermediate-risk prostate cancer, and clinical trials are ongoing. However, its success depends on a detailed understanding of the cellular and molecular mechanisms driving or hindering its antitumor efficacy [107].

On this subject, a noteworthy functional dichotomy essentially involves nitric oxide (NO). This mediator, produced not only by macrophages and endothelial cells in the tumor vasculature but also by the tumor cells, exhibits a complex role. There is a large body of evidence on the pro-cancerous role of NO at low concentrations, also through the promotion of aerobic glycolysis. Conversely, high NO concentrations promote its antitumor role. This dual role is influenced by various factors, such as the source of NO in TME, the characteristics of the TME-including local oxygen level- and NO generation rate [108]. In prostate cancer, the effect of ALA induced protoporphyrin IX-mediated PDT has been extensively studied. This treatment dramatically increases inducible NO synthase (iNOS/NOS2) activity, thus NO, resulting in a synergistic effect of resistance to cell death pathways (primarily apoptosis) while simultaneously promoting tumor growth, migration, and invasiveness [108,109].

Concerning PDT-induced NO-mediated cytoprotective molecular mechanisms, a further in vitro study on prostate cancer PC3 and LNCaP cells revealed key pathways in the activation of pro-survival/anti-apoptotic NF-κB and Yin Yang 1 (YY1). Concurrently, there was concomitant inhibition of anti-survival/pro-apoptotic factors and the metastasis suppressor RAF-kinase inhibitor protein (RKIP). These effects were observed at low PDT-induced NO levels, whereas at high PDT-induced NO levels, the diametrically opposite molecular events (inhibition of NF-κB and YY1 with RKIP activation) explain the antitumoral role of NO [110].

Far beyond these dichotomic mechanisms, PDT plays an essential antitumor role in prostate cancer through its classically induced cytotoxic events. These effects lead, in turn, to the activation of various cell death mechanisms. Specifically, in primary prostate epithelial cells cultured from prostate cancer patient samples, the in vitro cytotoxic effects of porphyrin-based PDT are characterized by a ROS-dependent reduction in cell viability and colony-forming ability, along with DNA damage and subsequent induction of both autophagy and necrosis, with apoptosis being less prominent as cell death mechanism [111]. Furthermore, in human prostate cancer PC-3 cells treated with Pheo-mediated PDT, an anti-invasive and anti-metastatic potential is observed via the inhibition of EMT and downregulation of MMPs [112].

However, aside from the well-known imbalance caused by massive ROS generation, PDT-induced tumor-killing effects are also significantly associated with the suppression of antioxidant signaling. This redox homeostasis disruption represents a promising new molecular target in anticancer therapy [113].

In a comparative study on the therapeutic efficacy of Pheo-mediated PDT in prostate cancer cells, a 75% rate of apoptotic cells was observed. This apoptosis resulted from PDT-induced increased levels of ROS and malondialdehyde, as well as the downregulation of SOD and decreased levels of GSH and CAT [114]. These antitumoral molecular mechanisms have recently been reproduced in PC3 cells using an innovative PDT model involving quaternized derivatives (Q-Zn1c, Q-Zn2c) [115]. Beyond apoptosis, autophagy-induced necroptosis has emerged as another mechanism of cell death in PC3-cells treated with MB-PDT. This was molecularly revealed by increased levels of LC3 (a marker of autophagy), and elevated phospho-mixed lineage kinase domain-like pseudokinase (a marker of necroptosis). The involvement of ROS in this process was further supported by higher lipidic peroxidation as well as by the downregulation of CAT and total antioxidant potential [116].

A new therapeutic frontier for prostate cancer may involve the use of PDT in functional synergy with the established antitumor efficacy of androgen deprivation therapy in castration-sensitive prostate cancer [117].

In androgen-dependent prostate cancer, Pheo-PDT has demonstrated the ability to induce G0/G1 cell cycle arrest and exhibit anti-migratory and anti-invasive properties, contributing to its anti-metastatic effects. This antitumor potential is ERS-dependent and attributable to the rapid Pheo-PDT-induced ROS generation [118]. Furthermore, similar cytotoxic and antitumor effects of Pheo-PDT have been confirmed in vitro on androgen-independent metastatic prostate cancer cell lines, such as DU-145 and C4-2 [119].

In recent years, the anti-cancer efficacy of phthalocyanine derivative-based PDT has been evaluated in prostate cancer DU145 and BPH-1 cells through non-canonical mechanisms. It has been identified that these derivatives can alter the activity of several signaling pathways, including PI3K, JNK, ERK1/2 and notch homolog protein 1(Notch1) as an alternative anticancer mechanism to the traditional ROS generation. This non-canonical notion is supported by the significant roles these pathways play in regulating apoptosis, proliferation, cell cycle, carcinogenesis, angiogenesis, metastasis, and immunomodulation [120].

Highlights:

- PDT-induced activation of iNOS was identified as a key cytoprotective and thus protumoral mechanism in prostate cancer cells. Interestingly, these events only occur at low PDT-induced NO levels, whereas at high PDT-induced NO levels, the antitumor regulatory role of NO prevails.
- Necroptosis and autophagy are PDT-induced cell death mechanism alternatives to apoptosis, which is well represented in prostate cancer. Nevertheless, when induced, apoptosis remains a phenomenon that is closely related to PDT-induced ROS generation and mitochondrial involvement.
  PDT-influenced cell death-related pathways:
  Apoptosis: iNOS/NOS2/NO/RKIP.
- In a non-canonical manner, some PDT approaches act in an antitumor mode by regulating signaling pathways as an alternative to classical ROS generation.

## 13. Bladder Cancer (BC)

BC is the leading malignancy of the urinary tract and, according to the most recent epidemiological data, ranks as the tenth most common cancer and thirteenth most common cause of cancer-related mortality worldwide [121,122]. In a clinical setting, PDT represents a pioneering approach for urothelial carcinoma. PDT holds promise for use in both adjuvant and neoadjuvant settings to reduce local recurrence and has potential for integration with other well-established therapeutic regimens [122].

However, despite its clinical promise, there is, to date, a lack of comprehensive understanding regarding the molecular and cellular mechanisms underlying the efficacy of PDT in this malignancy.

Much of the scientific attention is on elucidating the various PDT-elicited cell death pathways. Similar to findings in other neoplasms, preliminary evidence in BC suggests that apoptosis and autophagy may play functionally opposite roles.

Chlorophyllin f-based PDT in human BC 5637 and T24 cells has demonstrated significant pro-apoptotic and autophagic effects. The autophagic response, evidenced by the upregulation of the autophagy-related protein Beclin1 and LC3-I to LC3-II conversion, is thought to contribute to tumor resistance [123]. Additional preliminary evidence supports this functional dualism of apoptosis and autophagy in BC, involving other molecular actors such as autophagy-related enzyme 3. In contrast, apoptosis induction appears to be driven by massive ROS production alongside inhibited SOD activity [124,125].

An even more interesting aspect of cellular response involves the dysfunction of the autophagic process, which is believed to trigger necrosis as the main ROS-dependent cell death mechanism (80%), while significantly suppressing apoptosis (0.68%) [126].

Highlights:

- Various PDT strategies in BC induce apoptosis and necrosis as major pathways of cell death.
- Autophagy is ascribed a putative role as a cancer survival mechanism.
- According to current evidence, it is still unclear whether PDT-induced ROS generation induces apoptosis or necrosis as the major cell death mechanism in BC.

## 14. Cervical Cancer

Cervical cancer is the fourth most common cancer among women worldwide. However, despite persistent geographical disparities, epidemiological data from the last two decades offer some reassurance due to the implementation of cytological screening programs [127]. In the current era of promising new therapeutic approaches for this neoplasm, PDT plays a prominent clinical role, especially in the treatment of early-stage cervical cancer. PDT is also correlated with higher rates of human papillomavirus (HPV) eradication [128,129].

A substantial body of in vitro evidence identifies apoptosis as the primary mechanism of cell death induced by various PDT approaches in cervical cancer cells and epithelial cells transfected with high-risk HPV [130,131,132,133]. This apoptotic activation is primarily driven by ROS generation through simultaneous type I and type II photodynamic reactions, with type II reactions likely playing a predominant role [132]. Apoptosis predominantly involves the mitochondrial pathway, characterized by a transient upregulation of caspase-9 and a more sustained caspase-3 activation [130]. Moreover, PDT is associated with the upregulation of several oncosuppressors, such as p53, the CDK inhibitor p21, and retinoblastoma-associated protein 48 [131]. Alongside apoptosis, PDT also induces the downregulation of Notch-1, leading to the suppression of its downstream pathways, such as NF-κB and VEGF-A [134].

In addition to apoptosis, which is also ERS-dependent, preliminary evidence suggests that ALA-PDT-induced autophagy-likewise ERS-dependent-may contribute to PDT resistance in HeLa cells. The molecular basis for this dual activation involves the upregulation of the Ras/Raf/MEK/ERK pathway and the concomitant downregulation of the PI3K/AKT/mTOR pathway [135,136]. Additionally, necrosis also plays a role in the PDT-induced cell death pathways, affecting both HPV-immortalized and non-HPV-immortalized cervical cancer-derived cell lines [137].

Another field worth investigating is the influence of PDT on epigenetics and the subsequent modulation of alarmins, which play a key role in inducing antitumor immunity. Preliminary evidence suggests that, in HPV-positive cervical cancer cells, ALA-PDT downregulates miR-34a. Such downregulation leads to the upregulation of HMGB1, which in turn enhances antitumor immunity by increasing the percentage of mature DCs in peripheral blood and by elevating levels of proinflammatory cytokines such as IL-6, IL-12, IL-18, IFN-α, and TNF-α. In this immune setting, HPV viral load seems to play a key role in ALA-PDT-mediated modulation of the miR-34a/HMGB1 axis [138].

Additionally, in cervical intraepithelial neoplasia grade 2, ALA-PDT also promotes local immune infiltration of cytotoxic CD8 + T cells expressing GrzB, which can influence prognosis [139].

Highlights:

- In cervical cancer cells, PDT-induced apoptosis is the main mechanism of cell death and is consequent to both massive ROS generation and the upregulation of oncosuppressors p53 and RbAp48 as well as cell cycle inhibitors. In contrast, some investigations suggest that necrosis is the main mechanism of cell death, even in non-HPV-immortalized cervical cancer cells.
- PDT-influenced cell death-related pathways:
  Intrinsic apoptosis: ROS/Cyt-c/APAF1/caspase-9/caspase-3.
  Autophagy: Ras/Raf/MEK/ERK and PI3K/AKT/mTOR.
- There is preliminary evidence of the autophagic process as a PDT-resistance mechanism in cervical cancer cells.
- Very promising and deserving of further investigation is the role of ALA-PDT in promoting antitumor immunity both by upregulating HMGB1 alarmin levels and by directly targeting CD8 + T-cells.

## 15. Ovarian Cancer

Ovarian cancer has the highest mortality rate among the gynecological cancers in the world and is the second most common gynecological cancer in the United States. Its insidious clinical course results in more than half of all advanced or metastatic diagnoses [140]. In recent years, there has been increasing interest in the use of PDT for ovarian cancer, particularly in the context of a detailed cytomolecular characterization of the TME and emerging therapeutic strategies [141].

Recent research has focused on elucidating the resistance mechanisms directly or indirectly induced by PDT, especially in cancer cells that are already resistant to other canonical treatments. Preliminary evidence suggests the importance of intracellular PS accumulation for the cytotoxic effects of PDT. Furthermore, the PS accumulation level is directly proportional to the sensitivity of ovarian cancer cells to PDT [142].

**Figure 1:**
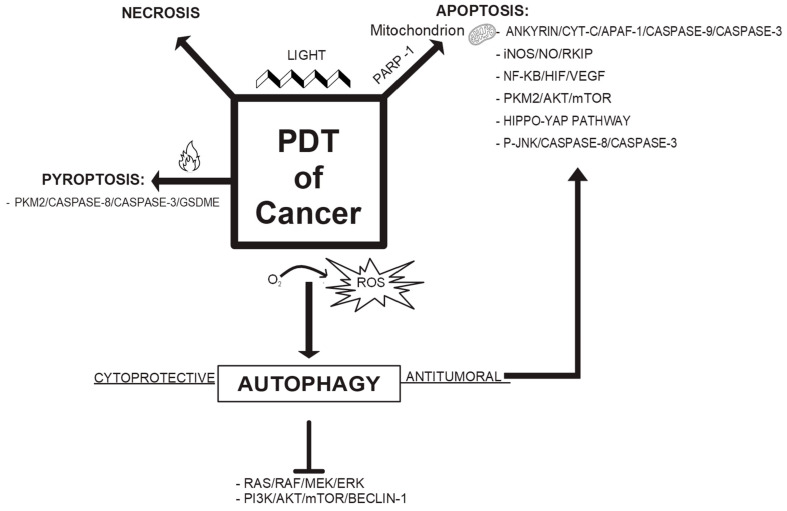
A comprehensive overview of the signaling pathways governing various cell death processes that are PDT-modulated. Abbreviations: cytochrome c (CYT-C), apoptotic protease activating factor-1 (APAF-1), nitric oxide (NO), inducible NO synthase (iNOS), rapidly accelerated fibrosarcoma (RAF)-kinase inhibitor protein (RKIP), nuclear factor kappa-light-chain-enhancer of activated B cells (NF-kB), hypoxia-inducible factor (HIF), vascular endothelial growth factor (VEGF), pyruvate kinase M2 (PKM2), protein kinase B (AKT), mechanistic target of rapamycin (mTOR), Hippo-Yes-associated protein-1 (HIPPO-YAP), phosphorylated c-Jun N-terminal kinase (p-JNK), N-gasdermin E (GSDME), rat sarcoma (RAS), rapidly accelerated fibrosarcoma (RAF), mitogen-activated protein kinase kinase (MEK), extracellular signal-regulated kinase (ERK), phosphatidylinositol 3-kinase (Pi3K).

In doxorubicin-resistant ovarian cancer cells, the activation of the c-methylnitronitrosoguanidine HOS transforming gene (MET)/PI3K/AKT signaling pathway is a putative mechanism of resistance to Pheo-PDT. Specifically, this signaling was responsible for overexpression of breast cancer resistance protein/ATP-binding cassette subfamily G member (ABCG2), which leads to the extracellular export of Pheo, thus reducing its cytotoxic effects [143]. Another emerging mechanism of tumor cell survival to PDT involves the PDT-induced HSP27 overexpression, which inhibits active apoptosome formation [144]. Apoptosis is the main PDT-induced cell death mechanism in ovarian cancer cells, closely dependent on down-regulation of nuclear factor erythroid 2-related factor 2 (Nrf2)-ABCG2 or Nrf2-HO-1 antioxidant signaling, leading to massive ROS generation [145,146,147]. Consequently, lysosomes become the primary targets, with disruption of their membrane integrity. This disruption results in the cytoprotective downregulation of the proton pump ATPase H + transporting V1/T cell immune regulator 1, and the consequent degradation of most intralysosomal acid hydrolases, aiming to prevent their destructive activity on other cellular structures [148] (Table 1) (Figure 1).

Highlights:

- In ovarian cancer, non-negligible tumor cell survival mechanisms are ascribed to the activation of specific pathways, such as c-MET/PI3K/AKT signaling, as well as to the PDT-induced action of specific alarmins such as HSP27.
- Apoptosis is reported to be the main PDT-induced cell death mechanism via massive ROS production and simultaneous PDT-induced downregulation of antioxidant signaling pathways.
- PDT-influenced cell death-related pathways: apoptosis: Nrf2-ABCG2 or Nrf2-HO-1.

## 16. Discussion

### 16.1. General Highlights

#### 16.1.1. PDT-Induced Cytological Phenomena

Apoptosis: This innate process is the primary mechanism of cell death observed across various tumors. It is triggered by high concentrations of ROS. In certain cancers, such as prostate cancer and ovarian cancer, PDT promotes apoptosis by downregulating antioxidant signaling [113,114,116,145,147]. Apoptosis involves both intrinsic (mitochondrial and ER-mediated) and extrinsic pathways. However, in some tumors, such as esophageal cancer (Eca-109 cells), apoptosis is functionally supported by autophagy [71]. In acute promyelocytic leukemia, apoptosis is the predominant cell death mechanism compared to PDT resistance pathways [103].

Autophagy: Triggered by increased ROS and influenced by PS intracellular localization, autophagy can either support apoptosis as an additional cell death mechanism or act as a survival mechanism. In several cancers, including breast cancer, melanoma, CRC, osteosarcoma (OS), BC, and cervical cancer, autophagy plays a protective role by inhibiting apoptosis, which can lead to PDT resistance [14,26,84,92,123,135].

Necrosis: Characterized by cell membrane disintegration and the release of cytosolic contents into the extracellular space [111]. In many tumors, necrosis occurs alongside apoptosis as a significant PDT-induced and ROS-dependent cell death mechanism. In bladder cancer, for instance, impaired autophagy and suppressed apoptosis may shift necrosis to the primary PDT-induced cell death mechanism [126].

Necroptosis: Observed in OS and prostate cancer cells, where it serves as an alternative cell death mechanism to apoptosis and is functionally supported by autophagy [30,91,116].

Ferroptosis: Preliminary evidence suggests that ferroptosis is induced by PDT in skin cancer, pancreatic cancer, CCA, esophageal carcinoma, and OS cells [22,28,44,78,99].

Pyroptosis: A newly identified PDT-induced cell death mechanism recently observed in pancreatic cancer, esophageal cancer, and CRC [36,77,85].

Immunogenic Cell Death (ICD): PDT-induced ICD has been reported in breast cancer, skin cancer, and HCC [9,22,48]. ICD is part of PDT’s broader antitumor immunostimulatory role, involving macrophage activation, which leads to the phagocytosis and presentation of tumor antigens to CTLs [55].

Antitumor Immune Response: Seen in various cancers (pancreatic cancer, HCC, gastric cancer, esophageal cancer, CRC, cervical cancer), this response is primarily driven by the clonal expansion of CTLs and concurrent restriction of Tregs [37,48,65,69,88,139].

#### 16.1.2. PDT-Influenced Biochemical Phenomena

Anti-Warburg Effect: A reduction in aerobic glycolysis has been observed in esophageal cancer cells [76].

Antioxidant Signaling: This can be either upregulated, as seen in OS cells (indicating a resistance mechanism to PDT), or downregulated in OS cells, prostate cancer cells, and ovarian cancer cells [98,114,116,145,146,147,149].

#### 16.1.3. PDT and Nanomedicine

PDT is crucial in killing tumor cells and stimulating various immune responses related to apoptotic and necrotic tumor cells, as well as inflammatory cells [150]. Nanoparticle-based PDT plays a key role in targeting undetectable and metastatic tumors, overcoming drug resistance, and regulating the TME [151]. Nanomaterials can be applied to enhance the antitumor immunity of PDT [152]. Nanoparticles, including liposomes, polymers, metallic nanomaterials, and porous silicon nanoparticles, offer several advantages that enhance pharmacological efficacy. These features include improved tumor penetration, prevention of drug degradation, enhanced drug solubility, and the ability to be tailored for tissue-specific targeting [106,153]. However, a significant limitation is the challenge posed by immune system surveillance and blood vessel barriers, which can impede the delivery of nanoplatform-based PSs to tumor-specific tissues.

A promising approach to overcome this challenge is the use of biomimicry in nanomedicine. For example, targeting the inflammatory TME is beneficial, as it plays a role in various stages of tumor development, particularly in cancers such as HCC. Biomimicking nanoparticles, functioning as a “Trojan horse”, can effectively deliver PSs into the inflammatory microenvironment [55].

#### 16.1.4. PDT Limitations

Although PDT is considered a groundbreaking therapeutic approach in oncology, it does have several biochemical, biophysical, and clinical limitations. Biochemically, the effectiveness of oxygen-dependent PDT can be compromised by the hypoxic tumor environment, which is exacerbated by therapeutic oxygen consumption, thereby creating a detrimental cycle that promotes tumor growth.

From a biophysical perspective, the activation of many PSs requires short-wavelength light (400–700 nm), which limits the use of PDT to peripheral anatomical regions (such as the skin) or areas accessible via endoscopy. This limitation arises from the poor tissue penetration of light, making it challenging to target deep-seated tumors (such as osteosarcoma). Additionally, the optical properties of PSs can be diminished by aggregation-caused quenching when PSs are administered at high concentrations.

Clinically, there is a risk of phototoxicity due to the off-target distribution and accumulation of systemically administered PSs [106,153,154]. Another critical issue is the potential development of resistance to PDT. This resistance has been observed in various previously treated malignancies (such as osteosarcoma, acute promyelocytic leukemia, prostate cancer, breast cancer, cervical cancer, and ovarian cancer) and is often linked to PDT-induced activation of autophagic processes, which can contribute to cancer cell survival.

#### 16.1.5. PDT-Based Combination Therapies

PDT is just one of the many anticancer phototherapy modalities currently available and increasingly studied, including, among others, photothermal, photoimmune, photo-gas, and radiotherapeutic approaches [155]. The combination of two or more of these approaches is revolutionizing cancer treatment strategies, resulting in continuously improving therapeutic efficacy. For example, both PDT and photothermal therapy (PTT) are capable of eliciting an antitumor immune response, thus effectively cooperating with immunotherapy. Moreover, PDT and PTT enhance each other’s therapeutic efficacy. In fact, while PDT increases the sensitivity of tumor cells to PTT, the latter, through heat generation and increased blood flow, improves oxygen supply, which is crucial for the photodynamic therapeutic effect [156].

An important issue concerns the inability of PDT alone to stimulate an immune response strong enough to counterbalance the release of immunosuppressive cytokines by cancer cells. This highlights the need to combine PDT with other therapeutic strategies that boost antitumor host immunity.

A promising new therapeutic approach is the combination of PDT with cancer immunotherapy, including checkpoint blockade immunotherapy, adoptive cell therapy, and cancer vaccines. The goal of immune-based therapies is to “awaken” the host immune system to mount an effective antitumor response. However, a significant limitation is the poor immunogenicity of many tumors, which can result in non-responsiveness and therapeutic failure when these therapies are used alone.

In this context, PDT’s ability to stimulate both innate and adaptive antitumor immunity and induce ICD is particularly valuable. ICD is considered a crucial link between PDT and cancer immunotherapy. Specifically, enhancing ICD via the combination of local PDT treatment with systemic inhibition of the Programmed Death (PD)-1/Programmed Death-Ligand 1 (PD-L1) immune checkpoint (which are negative regulators of immune response) would clinically be associated with both regression of primary tumors and regression of distant metastases [157]. Further impetus for the development of such combination therapeutic strategies comes from the use of new nanotechnologies that ensure effective delivery of both PSs and immunotherapeutic agents to the TME [158,159].

Additionally, conventional therapies such as chemotherapy, radiotherapy, and PTT have also been recognized for their capacity to activate ICD [154]. An innovative and highly promising anticancer therapy that activates the antitumor host immunity is near-infrared (NIR) photoimmunotherapy (PIT). NIR-PIT is a photochemistry-based cancer therapy consisting of the injection of a conjugate of a near-infrared, water-soluble silicon-phthalocyanine derivative, IRdye700DX (IR700), and a monoclonal antibody targeting a specific tumor-associated antigen expressed on the surface of cancer cells. Subsequent local exposure to NIR light triggers a rapid and highly selective ICD of the targeted cancer cell, which in turn results in the rapid maturation of immature dendritic cells adjacent to dying cancer cells, thereby activating the antitumor host immunity through the priming of polyclonal tumor-infiltrating CD8 + T cells against the released tumor antigens [160]. Interestingly, beyond its cytotoxic effects limited to cancer cells, NIR-PIT can also be repurposed to target and deplete a wide repertoire of non-cancerous immune-suppressive cells, such as Tregs, myeloid-derived suppressor cells, and cancer-associated fibroblasts, further enhancing the antitumor host immunity. In this regard, the therapeutic synergy between NIR-PIT and other immune-activating treatments is highly promising [161].

Emerging nanomedicines offer a revolutionary perspective by addressing the limitations of PDT. These advanced nanomedicines can effectively co-deliver PSs, immunomodulators, and chemotherapeutics to targeted sites, paving the way for a new era of combination therapy in oncology [154]. Even more so in this context, the emerging concept of “ROS science” not only aims to investigate the chemical mechanisms and biological effects related to ROS but also seeks to advance the understanding of ROS-based nanotherapies in order to improve therapeutic outcomes [162].

### 16.2. Remarks and Future Goals

The growing understanding of the cytomolecular mechanisms being directly PDT-modulated aligns with its clinical application across various cancer types.

### 16.3. State of the Art

**Figure 2:**
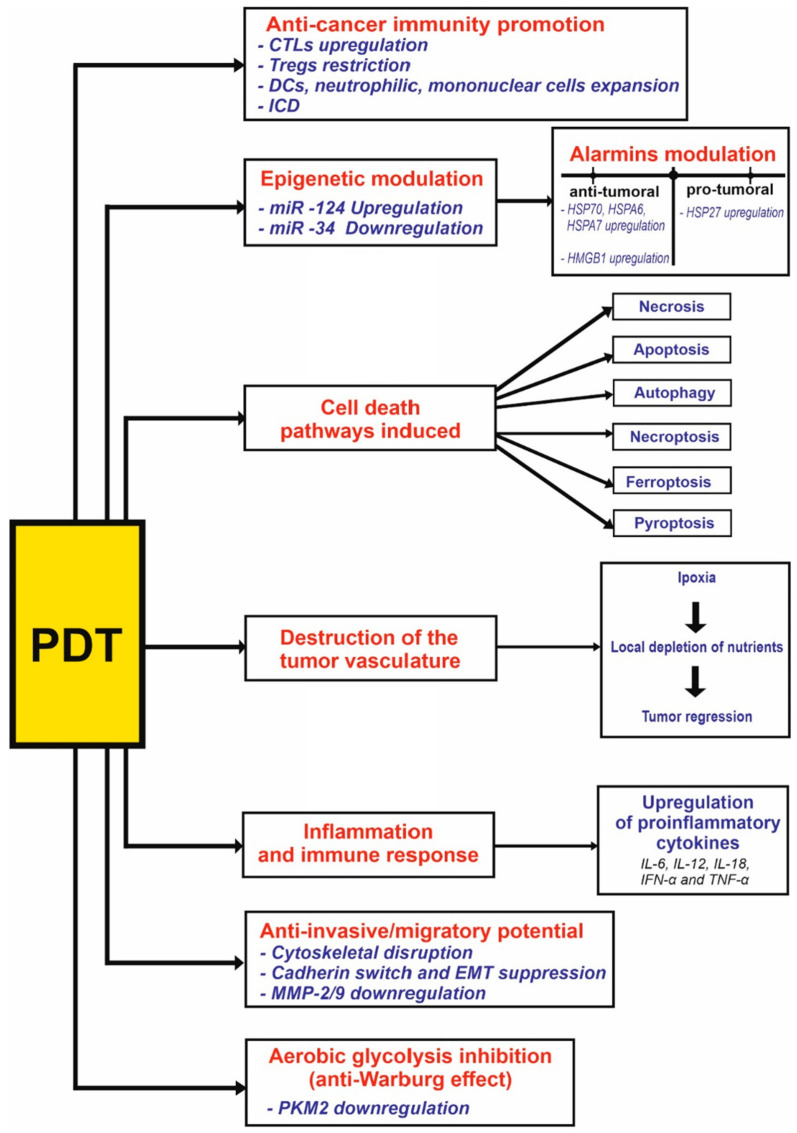
A schematic illustration of PDT’s impact on cytomolecular pathways. Abbreviations: photodynamic therapy (PDT), cytotoxic T lymphocytes (CTLs), dendritic cells (DCs), immunogenic cell death (ICD), microRNA (miR), heat shock protein (HSP) 70/A6/A7/27, High Mobility Group Box 1 (HMGB1), matrix metalloproteinase (MMP)-2/9, pyruvate kinase M2 (PKM2), Interleukin (IL)-6/12/18, interferon (IFN)-α, tumor necrosis factor (TNF)-α.

- PDT’s Dual Mechanism: PDT exerts its effects through both the destruction of tumor vasculature (indirect cytotoxicity) and the stimulation of the immune system (direct cytotoxicity and release of alarmins). This approach effectively counteracts key carcinogenic processes, including tumor neoangiogenesis and the suppression of antitumor immune responses.
- Immunogenic Potential: The emerging immunogenic properties of PDT, particularly through the activation of ICD, offer a promising avenue to address the limitations of cancer immunotherapy. This potential supports the development of novel combination therapies in nanomedicine.
- Autophagy and Resistance: PDT-induced autophagy has been identified as a significant mechanism of cancer cell survival across various tumor types, contributing to PDT resistance. Understanding the signaling pathways involved—whether cytoprotective or pro-apoptotic—is a critical area for further research. This knowledge could lead to more effective strategies for targeting PDT resistance in precision medicine (Figure 2).

## Figures and Tables

**Table 1:** Schematization of cytochemical events and related signaling pathways being involved in each PDT-targeted tumor. Abbreviations: Ribonucleic acid (RNA), endoplasmic reticulum (ER), Rat sarcoma (Ras), B-cell lymphoma 2 Homology 3 interacting-domain death agonist (Bid), B-cell lymphoma 2-associated X protein (Bax), cytochrome c (Cyt-c), apoptotic protease activating factor-1 (APAF-1), mechanistic target of rapamycin (mTOR), Hippo-Yes-associated protein-1 (HIPPO-YAP), Krueppel-like factor 10 (KLF10), epidermal growth factor receptor (EGFR), protein kinase RNA-Like ER kinase (PERK), phosphorylation of the eukaryotic initiation factor 2 (p-EIF), CCAAT-enhancer-binding protein homologous protein (CHOP), mitogen-activated protein kinase (MAPK), immunogenic cell death (ICD), nuclear factor kappa-light-chain-enhancer of activated B cells (NF-kB), hypoxia-inducible factor 1-alpha (HIF-1α), vascular endothelial growth factor (VEGF), phosphatidylinositol 3-kinase (Pi3K), protein kinase B (AKT), pyruvate kinase M2 (PKM2), p38 protein (p38), reactive oxygen species (ROS), caspase 3 (CASP3), N-gasdermin E (GSDME), cellular myelocytomatosis oncogene (c-myc), nuclear enriched abundant transcript 1 (NEAT1), microRNA (miR), Ras-related C3 botulinum toxin substrate 1 (Rac1), p21 protein (p21), Lin-11, Isl1, and MEC-3 (LIM), c-Jun N-terminal kinase (JNK), inositol-requiring enzyme 1 α (IRE1α), X-box binding protein 1 (XBP1), truncated Bid (tBid), nuclear factor erythroid 2-related factor 2 (Nrf2), adenosine triphosphate-binding cassette subfamily G member (ABCG2), eme-oxygenase 1 (HO-1).

Tumors	PDT-Influenced Cytological and Biochemical Events	Molecular Pathways Involved
Breast cancer [9,14]	Apoptosis, necrosis, autophagy, ICD	Intrinsic apoptosis: Bid/Bax/Cyt-c/APAF-1/caspase-9. Autophagy: mTOR/ Beclin-1.
Skin cancer [22,23,25,26]	Apoptosis, necrosis, autophagy, ICD	Apoptosis: mitochondria-dependent Autophagy: RIPK1, JNK and PI3K/AKT/mTOR
Pancreatic cancer [11,28,29,30,31,36,37]	Apoptosis, necrosis, ferroptosis, necroptosis, pyroptosis, fibroblast depletion, neoangiogenesis and vasculogenic mimicry inhibition	Apoptosis - Intrinsic pathway. - Hippo-YAP pathway.
Cholangiocarcinoma [42,43,44,45]	Apoptosis, autophagy, ferroptosis	Extrinsic apoptosis: procaspase-8/caspase-8/Caspase-3. Transcriptional regulation: KLF10/EGFR axis. Autophagy: PERK)/(p-EIF)2α/CHOP pathway.
Hepatocellular carcinoma [48,49,50,52,56]	Apoptosis, necrosis, ICD, clonal expansion of peripheral blood mononuclear cells	Apoptosis: MAPK-signaling pathway. ICD: macrophage phagocytosis/processing and exposure of tumor-associated antigens/antigen-specific T cell response.
Gastric cancer [60,61,62,63,64,65,66]	Apoptosis, autophagy, necrosis, CTLs clonal expansion and Tregs restriction	Apoptosis - Mitochondria-mediated: ankyrin repeat domain 1/Cyt-c/caspase-9/caspase-3. - ER-mediated: glucagon receptor/junctional sarcoplasmic reticulum protein 1/HSPs.
Esophageal cancer [68,69,71,72,73,74,75,76,77,78]	Apoptosis, autophagy, pyroptosis, Tregs inhibition, anti-Warburg effect, EMT suppression	Apoptosis: NF-κB/HIF-1α/VEGF and PI3K/AKT/mTOR signaling. Pyroptosis: PKM2/caspase-8/caspase-3/GSDME. EMT: PI3K/AKT/mTOR. Anti-Warburg effect: PKM2 downregulation.
Colorectal cancer [81,82,83,84,85,87,88]	Apoptosis, pyroptosis, autophagy, CTLs activation, cytoskeletal anti-migratory reorganization, anti-oncogenic epigenetic regulation, EMT suppression	Intrinsic apoptosis: p38 MAPK/caspase-9/caspase-3. Pyroptosis: ROS/p38/CASP3/GSDME. Epigenetic regulation: C-myc/NEAT-1/miR-124. Cytoskeletal reorganization: Rac1/p21 (Rac1)-activated kinase 1/LIM domain kinase 1/cofilin.
Osteosarcoma [91,92,93,94,95,96,97,98,99]	Apoptosis, necroptosis, autophagy, antioxidant signaling upregulation	Extrinsic apoptosis: ROS-JNK. ER-mediated apoptosis: PI3K/AKT/mTOR. Autophagy: ROS-JNK. Antioxidant signaling: inositol-requiring enzyme 1 α (IRE1α)-XBP1.
Blood cancer [101,102,103,104,105]	Apoptosis, autophagy, necrosis	Apoptosis: TRAIL/TRAIL-receptor system/caspase-8. - Intrinsic: P-JNK/caspase-9/caspase-3. - Extrinsic: caspase-8/tBid/caspase-3.
Prostate cancer [108,109,110,113,116,118,120]	Apoptosis, necroptosis, autophagy, EMT suppression, antioxidant signaling downregulation	Apoptosis: iNOS/NOS2/NO/RKIP
Bladder cancer [123,124,125,126]	Apoptosis, necrosis, autophagy, antioxidant signaling downregulation	Autophagy: beclin-1/autophagy-related enzyme 3
Cervical cancer [130,132,134,135,136,138,139]	Apoptosis, necrosis, autophagy, antitumor immunity (CTLs an DCs stimulation)	Intrinsic apoptosis: ROS/Cyt-c/APAF1/caspase-9/caspase-3. Autophagy: Ras/Raf/MEK/ERK and PI3K/AKT/mTOR. Epigenetic regulation: miR-34a/HMGB1.
Ovarian cancer [143,144,145,147,148]	Apoptosis, antioxidant signaling downregulation	Apoptosis: Nrf2-ABCG2 or Nrf2-HO-1

## Data Availability

Not applicable.
